# Supplementary material for: Animacy Processing in Autism: Event-Related Potentials Reflect Social Functioning Skills
Source: Brain Sci. 2023 Nov 29;13(12):1656. doi: 10.3390/brainsci13121656 (PMC10742338; doi:10.3390/brainsci13121656)
Supplement: Supplementary file 1 [file brainsci-13-01656-s001.zip › Table S2.pdf]

Table S2. Mean amplitudes and latencies per Group across the six electrode clusters, i.e. left/right frontotemporal, anteriofrontal and occipital brain regions. *significant Group by Animacy interactions ($p < 0.05$) appear in bold

		Animate		Inanimate	
		**Autistic**	**Controls**	**Autistic**	**Controls**
**Left frontotemporal cluster (FFT7H, FT7, FTT7H)**					
Amplitude	N50	−2.0 (2.9)	−1.0 (1.1)	−1.9 (1.8)	−0.7 (0.9)
	P100	0.2 (1.7)	0.3 (1.2)	0.4 (1.0)	0.2 (0.8)
	N200	−2.0 (2.2)	−1.4 (1.5)	−1.7 (1.6)	−1.0 (1.2)
	P300	1.4 (2.1)	1.7 (1.6)	1.3 (1.9)	1.2 (1.5)
	SPW	3.0 (6.5)	3.0 (5.1)	1.7 (3.2)	2.5 (4.1)
	SNW	−1.3 (2.1)	−5.6 (6.1)	−1.7 (2.7)	−4.7 (4.6)
Latency	N50	83.0 (21.1)	78.8 (19.7)	88.8 (22.5)	71.9 (26.2)
	P100	117.1 (30.8)	113.2 (21.9)	130.4 (50.6)	116.9 (24.6)
	N200	191.4 (20.6)	174.1 (15.6)	187.0 (32.6)	179.2 (18.9)
	P300	427.2 (73.9)	444.9 (47.2)	432.4 (50.8)	441.7 (32.6)
	SPW	838.2 (160.2)	844.7 (136.9)	873.4 (187.5)	833.7 (128.2)
	*SNW	1435.8 (262.5)	1666.5 (215.5)	1552.6 (293.9)	1588.8 (295.6)
**Right frontotemporal cluster (FFT8H, FT8, FTT8H)**					
Amplitude	N50	−0.9 (1.9)	−1.8 (2.3)	−1.2 (2.1)	−1.3 (1.4)
	P100	1.3 (2.6)	0.2 (1.2)	0.3 (1.9)	0.2 (0.8)
	N200	−1.4 (1.8)	−1.9 (1.9)	−1.7 (2.8)	−1.5 (1.3)
	P300	1.8 (3.4)	0.6 (1.3)	0.2 (1.3)	0.2 (1.2)
	SPW	4.9 (6.6)	2.3 (2.6)	2.9 (1.8)	0.2 (1.1)
	SNW	−0.9 (4.5)	−5.8 (2.4)	−1.5 (2.5)	−4.1 (6.0)
Latency	N50	56.0 (34.1)	58.3(17.9)	63.8 (22.8)	57.4 (20.8)
	P100	51.0 (10.3)	43.8 (15.8)	46.6 (14.5)	41.4 (18.6)
	N200	111.6 (20.3)	119.8(27.2)	100.8 (13.8)	114.1 (25.6)
	P300	252.8 (26.5)	240.9 (32.5)	246.8 (22.9)	250.5 (38.1)
	SPW	1096.4 (264.4)	881.3(250.6)	1071.4 (238.1)	854.2 (236.0)
	SNW	487.6 (78.9)	801.7 (10.2)	526.4 (95.6)	861.3 (229.0)
**Left anteriofrontal cluster (AFP1, AFF1H, AFF5H, AF3, AF7, AFF3H)**					
Amplitude	N50	−1.5 (3.2)	0.6 (0.7)	−0.25 (1.5)	0.3 (0.7)
	*N100	−0.6 (0.4)	−1.3 (0.6)	−1.9 (0.9)	−1.0 (0.5)
	P100	1.3 (5.9)	0.5 (1.1)	−0.5 (1.8)	0.2 (1.1)
	P300	3.5 (7.4)	0.4 (1.3)	0.7 (2.5)	0.6 (1.4)
	SPW	1.4 (1.3)	0.8 (0.6)	0.8 (2.6)	0.4 (2.6)
	SNW	−2.4 (4.4)	−5.8 (3.3)	−1.7 (2.2)	−4.7 (4.1)
Latency	N50	35.4(19.7)	38.4 (9.5)	45.4 (22.7)	37.9 (11.1)
	N100	97.6(44.5)	92.5 (19.1)	129.8 (27.2)	93.0 (19.2)
	P100	138.8(42.2)	143.9 (17.4)	180.4 (46.1)	149.0 (19.8)
	P300	363.3(6.9)	358.7(39.7)	386.6 (59.2)	372.2 (35.4)
	SPW	1702.8(389.6)	1715.7 (203.6)	1723.6 (327.2)	1710.5 (262.1)
	SNW	1403.8 (299.8)	1577.5 (264.5)	1601 (234.1)	1539.2 (230.9)
**Right anteriofrontal cluster (AFF6H, AFP2, AFF2H, AF4, AF8, AFF4H)**					
Amplitude	N50	−1.6 (2.8)	0.4 (0.6)	−0.3 (0.5)	0.2 (0.4)
	N100	−0.2 (2.6)	−1.2 (0.8)	−1.7 (1.9)	−0.9 (0.8)
	P100	1.7 (3.8)	0.4 (0.9)	−0.1 (0.9)	0.1 (0.7)
	P300	2.8 (6.1)	0.2 (0.4)	0.5 (1.4)	0.5 (0.9)
	SPW	4.5 (6.7)	2.9 (1.1)	2.3 (1.8)	2.6 (1.4)

	SNW	−1.2 (0.9)	−5.0 (4.2)	−1.3 (1.9)	−4.1 (3.7)
Latency	N50	37.2 (10.6)	41.1 (10.9)	33.4 (10.8)	39.9 (11.0)
	N100	82.6 (17.6)	91.4 (16.1)	90.2 (15.3)	93.3 (19.8)
	P100	151.2 (17.5)	141.2 (15.9)	159.2 (21.6)	144.0 (20.6)
	P300	363.8 (17.7)	364.5 (35.4)	374.2 (41.4)	370.7 (30.7)
	SPW	728.4 (199.2)	740.2 (138.1)	755.4 (83.8)	742.2 (161.4)
	SNW	1574.2 (253.6)	1496.5 (242.4)	1581.4 (193.7)	1543.8 (253.3)
**Left occipital cluster (O1, I1, OI1H)**					
Amplitude	P50	4.1 (2.8)	1.7 (2.1)	4.1 (5.6)	1.6 (1.8)
	P100	3.8 (2.9)	1.8 (2.1)	2.9 (4.9)	1.4 (1.9)
	P200	2.9 (3.2)	1.7 (2.3)	2.2 (3.5)	1.1 (2.2)
	N200	−0.3 (2.8)	−0.1 (1.5)	−0.8 (2.2)	−0.2 (1.7)
	P300	2.9 (2.9)	1.5 (1.9)	2.6 (3.5)	0.9 (1.7)
	N400	−3.6 (7.5)	−2.2 (1.5)	−2.2 (3.6)	−1.7 (1.4)
	SPW	3.5 (2.3)	6.4 (5.6)	1.9 (2.7)	5.1 (4.6)
Latency	P50	77.6 (42.7)	67.2 (18.9)	80.0 (35.2)	76.2 (21.1)
	P100	147.8 (40.1)	166.5 (21.4)	143.8 (24.7)	165.1 (23.5)
	P200	225 (38.2)	219.2 (15.3)	216.4 (29.2)	216.4 (14.9)
	N200	256.4 (22.9)	262.7 (22.6)	255.0 (24.4)	257.1 (22.6)
	*P300	354.4 (24.1)	324.5 (45.2)	308.6 (24.4)	347.9 (42.8)
	N400	693.2(259.3)	696.1 (169.5)	661.2 (191.7)	673.4 (168.5)
	SPW	1153.6 (438.4)	1468.2 (322.8)	1208.0 (490.1)	1493.2 (321.5)
**Right occipital cluster (O2, I2, OI2H)**					
Amplitude	P50	3.3 (2.3)	1.8 (2.1)	3.6 (4.1)	1.6 (2.1)
	P100	0.2 (1.7)	0.5 (1.2)	0.8 (2.7)	0.2 (0.8)
	P200	3.4 (3.1)	2.3 (2.4)	3.0 (3.7)	1.9 (2.2)
	N200	−0.4 (1.4)	−0.1 (1.2)	−0.2 (0.7)	0.1 (1.1)
	P300	3.3 (1.1)	1.6 (1.8)	3.2 (1.9)	1.1 (1.3)
	N400	−0.2 (3.0)	−0.2 (0.6)	−1.3 (1.5)	−0.1 (0.6)
	SNW	−5.7 (4.6)	−3.2 (2.4)	−2.9 (1.6)	−2.8 (1.4)
Latency	P50	83.2 (25.1)	64.2 (21.6)	76.8 (30.9)	73.8 (23.2)
	P100	112.2 (30.4)	116.3 (22.8)	129.0 (26.2)	113.5 (23.8)
	P200	190.2 (22.6)	171.8 (21.2)	195.4(27.8)	187.2 (24.9)
	N200	257.4 (10.1)	265.8 (23.3)	251.8 (11.1)	269.5 (25.0)
	P300	314.0 (20.2)	297.2 (35.7)	315.6 (23.9)	291.8 (34.4)
	N400	393.6 (51.1)	397.0 (36.6)	414.0 (43.0)	397.6 (34.6)
	SNW	941.0 (126.0)	855.8 (175.0)	959.0 (150.4)	847.8 (165.8)

Footnote. SNW = Slow Negative Wave; SPW = Slow Positive Wave
