# Supplementary material for: Animacy Processing in Autism: Event-Related Potentials Reflect Social Functioning Skills
Source: Brain Sci. 2023 Nov 29;13(12):1656. doi: 10.3390/brainsci13121656 (PMC10742338; doi:10.3390/brainsci13121656)
Supplement: Supplementary file 1 [file brainsci-13-01656-s001.zip › Table S3.pdf]

Table S3. ERP values for each autistic participant in the ERP components that exhibited statistically significant Group by Animacy interactions.

Autistic participants (P)	Left frontotemporal cluster SNW latency		Left anteriofrontal cluster N100 amplitude		Left occipital cluster P300 latency	
	**animate**	**inanimate**	**animate**	**inanimate**	**animate**	**inanimate**
P#1	1711	1977	−0.76	−2.95	386	276
P#2	1643	1999	0.36	−3.62	345	324
P#3	1228	1540	−1.07	−1.99	380	361
P#4	1211	1193	−0.66	−1.91	378	331
P#5	1341	1545	−0.65	−1.03	363	297
P#6	1784	1488	−0.89	−1.58	338	298
P#7	1795	1483	−0.69	−1.98	314	296
P#8	1223	1068	−0.68	−1.36	366	310
P#9	1244	1708	−0.67	−1.88	348	290
P#10	1178	1525	−0.73	−1.30	326	303

Footnote. SNW = Slow Negative Wave
