# Supplementary material for: Animacy Processing in Autism: Event-Related Potentials Reflect Social Functioning Skills
Source: Brain Sci. 2023 Nov 29;13(12):1656. doi: 10.3390/brainsci13121656 (PMC10742338; doi:10.3390/brainsci13121656)
Supplement: Supplementary file 1 [file brainsci-13-01656-s001.zip › Table S4.pdf]

Table S4. Correlation coefficients and *p* values between each group's social comprehension scores and ERPs across the six electrode clusters (left/right frontotemporal, anteriofrontal and occipital brain regions) separately for the animate and inanimate trials.

		Animate		Inanimate	
		**Autistic**	**Controls**	**Autistic**	**Controls**
		**Left frontotemporal cluster (FFT7H, FT7, FTT7H)**			
Amplitude	N50	$r = -0.379$	$r = 0.073$	$r = -0.321$	$r = -0.232$
		$p = 0.280$	$p = 0.803$	$p = 0.366$	$p = 0.424$
	P100	$r = 0.480$	$r = 0.262$	$r = 0.503$	$r = 0.038$
		$p = 0.160$	$p = 0.366$	$p = 0.138$	$p = 0.896$
	N200	$r = -0.448$	$r = -0.479$	$r = -0.177$	$r = -0.240$
		$p = 0.194$	$p = 0.083$	$p = 0.626$	$p = 0.409$
	P300	$r = 0.612$	$r = 0.059$	$r = 0.599$	$r = -0.222$
		$p = 0.070$	$p = 0.842$	$p = 0.077$	$p = 0.445$
	SPW	$r = -0.393$	$r = -0.369$	$r = -0.884$	$r = -0.340$
		$p = 0.262$	$p = 0.194$	$p = 0.001$	$p = 0.235$
	SNW	$r = -0.144$	$r = 0.162$	$r = 0.381$	$r = -0.184$
		$p = 0.691$	$p = 0.581$	$p = 0.277$	$p = 0.528$
Latency	N50	$r = -0.127$	$r = -0.451$	$r = 0.080$	$r = -0.433$
		$p = 0.727$	$p = 0.105$	$p = 0.825$	$p = 0.122$
	P100	$r = -0.439$	$r = 0.155$	$r = 0.438$	$r = 0.081$
		$p = 0.204$	$p = 0.596$	$p = 0.206$	$p = 0.783$
	N200	$r = 0.074$	$r = -0.089$	$r = 0.017$	$r = -0.211$
		$p = 0.839$	$p = 0.761$	$p = 0.963$	$p = 0.469$
	P300	$r = -0.426$	$r = 0.132$	$r = -0.477$	$r = 0.101$
		$p = 0.219$	$p = 0.653$	$p = 0.163$	$p = 0.731$
	SPW	$r = -0.598$	$r = -0.016$	$r = -0.530$	$r = -0.024$
		$p = 0.068$	$p = 0.958$	$p = 0.115$	$p = 0.935$
	*SNW	$r = -0.531$	$r = -0.235$	$r = -0.618$	$r = -0.324$
		$p = 0.114$	$p = 0.418$	$p = 0.067$	$p = 0.258$
		**Right frontotemporal cluster (FFT8H, FT8, FTT8H)**			
Amplitude	N50	$r = -0.131$	$r = 0.239$	$r = -0.439$	$r = 0.284$
		$p = 0.717$	$p = 0.410$	$p = 0.204$	$p = 0.326$
	P100	$r = 0.104$	$r = 0.003$	$r = -0.564$	$r = -0.291$
		$p = 0.774$	$p = 0.993$	$p = 0.090$	$p = 0.313$
	N200	$r = -0.382$	$r = 0.062$	$r = -0.601$	$r = 0.353$
		$p = 0.276$	$p = 0.834$	$p = 0.066$	$p = 0.216$
	P300	$r = -0.443$	$r = 0.072$	$r = -0.453$	$r = 0.255$
		$p = 0.199$	$p = 0.807$	$p = 0.189$	$p = 0.379$
	SPW	$r = 0.460$	$r = 0.130$	$r = -0.456$	$r = 0.235$
		$p = 0.181$	$p = 0.658$	$p = 0.185$	$p = 0.420$
	SNW	$r = -0.233$	$r = -0.066$	$r = -0.577$	$r = 0.325$
		$p = 0.518$	$p = 0.823$	$p = 0.081$	$p = 0.257$
Latency	N50	$r = -0.301$	$r = 0.146$	$r = 0.614$	$r = 0.378$
		$p = 0.398$	$p = 0.618$	$p = 0.069$	$p = 0.183$
	P100	$r = -0.151$	$r = 0.288$	$r = -0.501$	$r = 0.100$
		$p = 0.677$	$p = 0.317$	$p = 0.140$	$p = 0.735$
	N200	$r = -0.080$	$r = 0.234$	$r = -0.224$	$r = 0.234$
		$p = 0.826$	$p = 0.414$	$p = 0.5333$	$p = 0.421$
	P300	$r = -0.352$	$r = 0.197$	$r = -0.125$	$r = 0.411$
		$p = 0.318$	$p = 0.499$	$p = 0.730$	$p = 0.145$

	SPW	$r = 0.258$	$r = -0.287$	$r = 0.054$	$r = 0.076$
		$p = 0.373$	$p = 0.320$	$p = 0.855$	$p = 0.796$
	SNW	$r = 0.478$	$r = 0.434$	$r = -0.305$	$r = 0.210$
		$p = 0.084$	$p = 0.121$	$p = 0.289$	$p = 0.472$
**Left anteriofrontal cluster (AFP1, AFF1H, AFF5H, AF3, AF7, AFF3H)**					
Amplitude	N50	$r = 0.048$	$r = -0.129$	$r = -0.379$	$r = 0.098$
		$p = 0.894$	$p = 0.660$	$p = 0.280$	$p = 0.739$
	*N100	$r = -0.363$	$r = 0.351$	$r = 0.407$	$r = 0.290$
		$p = 0.303$	$p = 0.218$	$p = 0.244$	$p = 0.314$
	P100	$r = 0.222$	$r = -0.298$	$r = -0.121$	$r = -0.070$
		$p = 0.537$	$p = 0.302$	$p = 0.739$	$p = 0.813$
	P300	$r = 0.521$	$r = 0.010$	$r = 0.411$	$r = 0.064$
		$p = 0.122$	$p = 0.973$	$p = 0.237$	$p = 0.827$
	SPW	$r = 0.280$	$r = -0.035$	$r = 0.243$	$r = 0.231$
		$p = 0.432$	$p = 0.904$	$p = 0.499$	$p = 0.427$
	SNW	$r = 0.395$	$r = 0.253$	$r = 0.420$	$r = 0.202$
		$p = 0.259$	$p = 0.382$	$p = 0.227$	$p = 0.488$
Latency	N50	$r = 0.201$	$r = -0.101$	$r = -0.079$	$r = -0.004$
		$p = 0.577$	$p = 0.730$	$p = 0.828$	$p = 0.988$
	N100	$r = 0.108$	$r = -0.507$	$r = -0.293$	$r = -0.095$
		$p = 0.766$	$p = 0.064$	$p = 0.411$	$p = 0.746$
	P100	$r = 0.109$	$r = -0.228$	$r = -0.240$	$r = -0.358$
		$p = 0.765$	$p = 0.434$	$p = 0.504$	$p = 0.209$
	P300	$r = -0.144$	$r = -0.312$	$r = -0.413$	$r = -0.075$
		$p = 0.692$	$p = 0.277$	$p = 0.235$	$p = 0.798$
	SPW	$r = 0.163$	$r = -0.058$	$r = 0.059$	$r = -0.015$
		$p = 0.654$	$p = 0.843$	$p = 0.872$	$p = 0.959$
	SNW	$r = 0.147$	$r = 0.255$	$r = 0.528$	$r = 0.167$
		$p = 0.686$	$p = 0.378$	$p = 0.117$	$p = 0.569$
**Right anteriofrontal cluster (AFF6H, AFP2, AFF2H, AF4, AF8, AFF4H)**					
Amplitude	N50	$r = 0.168$	$r = 0.055$	$r = -0.014$	$r = 0.234$
		$p = 0.643$	$p = 0.851$	$p = 0.970$	$p = 0.422$
	N100	$r = -0.239$	$r = 0.056$	$r = -0.003$	$r = -0.049$
		$p = 0.507$	$p = 0.849$	$p = 0.972$	$p = 0.869$
	P100	$r = 0.338$	$r = 0.010$	$r = 0.104$	$r = 0.042$
		$p = 0.339$	$p = 0.972$	$p = 0.774$	$p = 0.886$
	P300	$r = 0.257$	$r = -0.064$	$r = 0.137$	$r = -0.103$
		$p = 0.474$	$p = 0.828$	$p = 0.708$	$p = 0.727$
	SPW	$r = 0.239$	$r = 0.380$	$r = 0.465$	$r = 0.346$
		$p = 0.505$	$p = 0.180$	$p = 0.176$	$p = 0.226$
	SNW	$r = -0.144$	$r = 0.110$	$r = -0.178$	$r = 0.254$
		$p = 0.690$	$p = 0.707$	$p = 0.622$	$p = 0.380$
Latency	N50	$r = 0.1833$	$r = 0.130$	$r = 0.327$	$r = 0.107$
		$p = 0.613$	$p = 0.658$	$p = 0.357$	$p = 0.716$
	N100	$r = 0.106$	$r = -0.037$	$r = -0.272$	$r = 0.269$
		$p = 0.771$	$p = 0.900$	$p = 0.446$	$p = 0.353$
	P100	$r = 0.096$	$r = 0.104$	$r = -0.127$	$r = 0.183$
		$p = 0.793$	$p = 0.724$	$p = 0.726$	$p = 0.352$
	P300	$r = -0.537$	$r = 0.060$	$r = -0.340$	$r = -0.029$
		$p = 0.109$	$p = 0.839$	$p = 0.336$	$p = 0.920$
	SPW	$r = 0.168$	$r = 0.480$	$r = 0.176$	$r = -0.001$
		$p = 0.642$	$p = 0.082$	$p = 0.627$	$p = 0.997$

	SNW	*r* = 0.245	*r* = −0.083	*r* = 0.460	*r* = −0.167
		p = 0.495	*p* = 0.778	*p* = 0.181	*p* = 0.568
		**Left occipital cluster (O1, I1, OI1H)**			
Amplitude	P50	*r* = 0.562	*r* = 0.150	*r* = 0.416	*r* = 0.053
		p = 0.091	*p* = 0.608	*p* = 0.231	*p* = 0.857
	P100	*r* = 0.401	*r* = −0.013	*r* = 0.435	*r* = −0.436
		p = 0.251	*p* = 0.965	*p* = 0.209	*p* = 0.120
	P200	*r* = 0.495	*r* = 0.277	*r* = 0.402	*r* = −0.008
		p = 0.146	*p* = 0.339	*p* = 0.250	*p* = 0.980
	N200	*r* = 0.215	*r* = 0.422	*r* = 0.273	*r* = 0.370
		p = 0.552	*p* = 0.132	*p* = 0.446	*p* = 0.192
	P300	*r* = 0.599	*r* = 0.229	*r* = 0.397	*r* = −0.021
		p = 0.067	*p* = 0.430	*p* = 0.255	*p* = 0.945
	N400	*r* = −0.273	*r* = −0.310	*r* = −0.491	*r* = −0.153
		p = 0.446	*p* = 0.281	*p* = 0.149	*p* = 0.602
	SPW	*r* = 0.511	*r* = −0.028	*r* = 0.338	*r* = −0.218
		p = 0.131	*p* = 0.924	*p* = 0.340	*p* = 0.455
Latency	P50	*r* = 0.575	*r* = 0.110	*r* = 0.550	*r* = 0.082
		p = 0.082	*p* = 0.707	*p* = 0.100	*p* = 0.781
	P100	*r* = 0.475	*r* = 0.050	*r* = 0.558	*r* = −0.162
		p = 0.165	*p* = 0.865	*p* = 0.134	*p* = 0.580
	P200	*r* = −0.345	*r* = 0.412	*r* = 0.586	*r* = 0.241
		p = 0.329	*p* = 0.144	*p* = 0.075	*p* = 0.406
	N200	*r* = −0.112	*r* = 0.158	*r* = 0.025	*r* = −0.026
		p = 0.758	*p* = 0.590	*p* = 0.945	*p* = 0.929
	*P300	*r* = 0.385	*r* = −0.369	*r* = 0.500	*r* = 0.415
		p = 0.272	*p* = 0.194	*p* = 0.141	*p* = 0.140
	N400	*r* = −0.403	*r* = −0.024	*r* = −0.285	*r* = 0.118
		p = 0.249	*p* = 0.935	*p* = 0.424	*p* = 0.689
	SPW	*r* = −0.338	*r* = −0.074	*r* = 0.333	*r* = −0.138
		p = 0.340	*p* = 0.802	*p* = 0.346	*p* = 0.639
		**Right occipital cluster (O2, I2, OI2H)**			
Amplitude	P50	*r* = 0.497	*r* = 0.182	*r* = 0.390	*r* = 0.195
		p = 0.144	*p* = 0.534	*p* = 0.265	*p* = 0.507
	P100	*r* = 0.462	*r* = −0.261	*r* = −0.572	*r* = −0.504
		p = 0.179	*p* = 0.367	*p* = 0.084	*p* = 0.066
	P200	*r* = 0.602	*r* = −0.072	*r* = 0.466	*r* = −0.297
		p = 0.065	*p* = 0.808	*p* = 0.175	*p* = 0.302
	N200	*r* = 0.591	*r* = −0.271	*r* = −0.570	*r* = −0.144
		p = 0.072	*p* = 0.349	*p* = 0.086	*p* = 0.622
	P300	*r* = 0.469	*r* = 0.445	*r* = 0.400	*r* = 0.283
		p = 0.172	*p* = 0.111	*p* = 0.252	*p* = 0.326
	N400	*r* = 0.563	*r* = −0.126	*r* = −0.283	*r* = −0.224
		p = 0.090	*p* = 0.667	*p* = 0.429	*p* = 0.441
	SNW	*r* = 0.507	*r* = −0.228	*r* = 0.411	*r* = 0.114
		p = 0.135	*p* = 0.433	*p* = 0.238	*p* = 0.698
Latency	P50	*r* = −0.345	*r* = 0.114	*r* = 0.377	*r* = −0.249
		p = 0.328	*p* = 0.697	*p* = 0.282	*p* = 0.390
	P100	*r* = −0.096	*r* = 0.253	*r* = 0.486	*r* = 0.118
		p = 0.792	*p* = 0.384	*p* = 0.155	*p* = 0.688
	P200	*r* = 0.478	*r* = 0.147	*r* = 0.542	*r* = −0.045
		p = 0.162	*p* = 0.616	*p* = 0.106	*p* = 0.880

N200	*r* = −0.118	*r* = −0.472	*r* = 0.416	*r* = 0.282
	p = 0.746	*p* = 0.089	*p* = 0.231	*p* = 0.328
P300	*r* = −0.570	*r* = 0.221	*r* = 0.364	*r* = −0.344
	p = 0.085	*p* = 0.448	*p* = 0.302	*p* = 0.228
N400	*r* = 0.451	*r* = −0.185	*r* = 0.247	*r* = 0.323
	p = 0.191	*p* = 0.525	*p* = 0.492	*p* = 0.259
SNW	*r* = −0.037	*r* = −0.049	*r* = −0.196	*r* = −0.105
	p = 0.919	*p* = 0.867	*p* = 0.588	*p* = 0.722

Footnote. SNW = Slow Negative Wave; SPW = Slow Positive Wave
